# Drawing explicit phylogenetic networks and their integration into SplitsTree

**DOI:** 10.1186/1471-2148-8-22

**Published:** 2008-01-24

**Authors:** Tobias H Kloepper, Daniel H Huson

**Affiliations:** 1 Center for Bioinformatics ZBIT, Tübingen University, Sand 14, 72076 Tübingen, Germany

## Abstract

**Background:**

SplitsTree provides a framework for the calculation of phylogenetic trees and networks. It contains a wide variety of methods for the import/export, calculation and visualization of phylogenetic information. The software is developed in Java and implements a command line tool as well as a graphical user interface.

**Results:**

In this article, we present solutions to two important problems in the field of phylogenetic networks. The first problem is the visualization of explicit phylogenetic networks. To solve this, we present a modified version of the equal angle algorithm that naturally integrates reticulations into the layout process and thus leads to an appealing visualization of these networks. The second problem is the availability of explicit phylogenetic network methods for the general user. To advance the usage of explicit phylogenetic networks by biologists further, we present an extension to the SplitsTree framework that integrates these networks. By addressing these two problems, SplitsTree is among the first programs that incorporates *implicit *and *explicit *network methods together with standard phylogenetic tree methods in a graphical user interface environment.

**Conclusion:**

In this article, we presented an extension of SplitsTree 4 that incorporates explicit phylogenetic networks. The extension provides a set of core classes to handle explicit phylogenetic networks and a visualization of these networks.

## Background

Phylogenetic networks are graphs used for representing phylogenetic relationships between different taxa, and are usually employed when a tree representation does not suffice. There are many different types of phylogenetic networks and it is useful to distinguish between two main classes: *implicit *phylogenetic networks that provide tools to visualize and analyze incompatible phylogenetic signals, such as split networks [1,2], and *explicit *phylogenetic networks that provide explicit scenarios of reticulate evolution, such as hybridization networks [3-7], HGT networks [8] and recombination networks [9-19].

The software currently available for the calculation and analysis of explicit phylogenetic networks consists of a spread of basic implementations of algorithms developed to solve the computational task [6,14,16-18,20]. Most of the software is command line driven and an appealing visualization of the results is often lacking. It is essential to have a tool that allows both broad usage of the methods available to biologists, and better and further development of new methods.

SplitsTree is an application developed in our research group, originally aiming at the phylogenetic analysis of sets of splits. The newest version of SplitsTree [2] incorporates a variety of methods for the calculation, visualization and interpretation of phylogenetic trees and implicit phylogenetic networks. Two main advantages of SplitsTree are the graphical user interface (GUI) and the integration of algorithms via an interface driven class loader (plugins). In this article we present an extension to SplitsTree that enables the program to handle explicit phylogenetic networks. The extension solves two important problems: an efficient integration of explicit phylogenetic networks, and visualizing these networks.

## Results and Discussion

A *tree T *= (*V*, *E*) is a connected acyclic graph with vertex set *V *and edge set *E*. A vertex of degree one is called a *leaf *of *T *and the set of all leaves is called the *leaf set *of *T*. A *rooted tree T *= (*V*, *E*, *ϱ*) is a tree (*V*, *E*) that has exactly one distinguished vertex called the *root*, denoted *ϱ*. A rooted tree *T *has a natural ordering where *v *≤ *v'*, if *v *lies on the path from the root to *v'*. If *v *≤ *v'*, we say that *v *is an *ancestor *of *v' *and *v' *is a *descendant *of *v*. For any set of vertices *V*, a vertex *v *is called *minimal *with respect to *V *if for all *v' *in *V*, it holds that *v *≤ *v'*. For any edge *e*, we use *α *(*e*) and *β*(*e*) to denote the source and target of *e*. A *rooted phylogenetic X-tree *is a pair (*T*, *ν*), where *T *= (*V*, *E*, *ϱ*) is a rooted tree and *ν *: *X *→ *V *is a bijection from *X *to the leaf set of *T*. See [21] for more details.

**Definition 1 ***Let X be a set of taxa. A *rooted reticulate network *N *= *N *(*V*, *E*, *ν*) on *X is a connected, directed acyclic graph with vertex set V, edge set E and vertex labeling ν : X → V, such that:*

*1. there exists precisely one distinguished vertex ϱ called the *root;

*2. every vertex v *∈ *V is either a *tree vertex, *v *∈ *V*_*T*_, *that has exactly one ancestor, or a *reticulation vertex *r *∈ *V*_*R *_*that has exactly two ancestors;*

*3. every edge is either a *tree edge *leading to a vertex of indegree one or a *reticulation edge *leading to a vertex of indegree two; and*

*4. the set of *leaves *L (vertices with no descendants) consists only of tree vertices and is labeled by the set of taxa X, i.e. ν maps X bijectively onto L*.

It follows from these definitions that each reticulation vertex (or *reticulation*, for short) *r *∈ *V*_*R *_is contained in one or more cycles of the form *C *= (*r*, *p*(*r*), *w*_1_, *e*_1_, ..., *e*_*k*-1_, *w*_*k*_, *q*(*r*), *r*), with *w*_*i *_∈ *V *and *e*_*i *_∈ *E*\{*p*(*r*), *q*(*r*)} for all *i*. (Note that additionally, *r *can also be contained in one or more cycles that do not contain *p*(*r*) and *q*(*r*)). We say that two reticulations *r*, *r' *∈ *V*_*R *_are *dependent *if a cycle that contains both *r *and *r' *exists.

In graph theory, a *two-connected component *of a graph *G *is any maximal subgraph *G' *with the property that any two vertices *v *and *w *of *G' *are connected by two paths *p *and *p' *that share no vertices except for *v *and *w*. For any reticulation vertex *r*, let *p*^*r *^and *q*^*r *^denote the two associated reticulation edges.

Furthermore, let $v_{p}^{r}$ and $v_{q}^{r}$ denote the two ancestors of *r *with respect to *p*^*r *^and *q*^*r*^. The *lowest single ancestor lsa*(*r*) of a reticulation *r *is the minimum of all nodes in *V *that is connected to *r *by two paths *p *and *p' *that share no vertices except for *lsa*(*r*) and *r*.

### Algorithm

One important approach to drawing trees is the equal angle algorithm which was developed by Meacham (see [22]). The equal angle algorithm guarantees that no two edges intersect. Our algorithm for visualizing recombination networks is based on a generalization of the equal angle algorithm. The algorithms adds an ordering step at each vertex, that chooses an optimal ordering of the descending edges, that minimizes the number of crossings between reticulations edges and other edges. It can easily be altered to be used with any drawing algorithm for trees. We will start out with a description of the equal angle algorithm and will then define some basic properties. Finally, we will give solutions to minimize crossing edges in a drawing of a reticulate networks, and the optimal placement of reticulation vertices.

The equal angle algorithm is a recursive algorithm that starts at an internal vertex of a tree. For each subtree connected to the starting vertex, we appoint an angle proportional to the share of leaves it contains. In the next step, we assign to each subtree a sector of the circle of the size of the angle appointed to it and draw the edge to the subtree in the middle of the sector. We place the sector of the subtree in a way that it is centered at the end of the branch and the branch is pointing at the bisector of the angle. We then recurse to the starting vertex of the subtree and assign each newly discovered subtree its proportional share of the angle. Each subtree is than placed in the sector of the starting vertex. The recursion is repeated until we have appointed angles to each branch of the tree. The only modifications for rooted trees are the explicit start point (the root of the tree) and the use of a fraction of the cycle. For a detailed description of the algorithm, see [22].

The rooted equal angle algorithm is not directly applicable to a reticulate network since for each reticulation, we have to decide which of the reticulation edges we want to use for the drawing algorithm and either choice may be suboptimal. The idea behind our approach is to use neither of them. The *influence *of a reticulation upon the graph structure is bounded by the reticulation and its lowest single ancestor, therefore we decided to define an *auxiliary edge *between those two vertices and to use the auxiliary edges for the layout of the graph. When the algorithm reaches a node each descending edge is checked for its status (being either a tree-edge, an auxiliary-edge or a reticulation-edge) and only tree- and auxiliary-edges are incorporated into the process.

Through these modifications to the rooted equal angle algorithm, it is possible to visualize reticulate networks, but these visualizations are not very satisfying. To obtain an improved method, we will address two key problems. The first problem is the crossing of reticulation edges: even though it can not always be avoided, the number of such events should be minimized. The second problem is that the auxiliary edges are artifical edges and their optimal edge length must be determined. In the following, we will show solutions to these two problems.

#### Minimizing crossing edges

An edge crossing another one is an undesirable event in drawing a graph. It is well known that solving this problem is, in general, computationally hard [23]. The equal angle algorithm ensures that we only have to deal with reticulation edges crossing other edges. Furthermore, the construction of the auxiliary edges implies that edges that can be crossed by the reticulation edges are descendent edges of the lowest single ancestor of the reticulation. The optimization starts at the root of the networks and optimizes the arrangement of the directly descending vertices. It then continues the optimization iteratively at each directly descending vertex in the order given and keeps going until it has optimized all placements.

Let $V_{v}^{T}$ be the set of tree vertices directly below a vertex *v *and let $V_{v}^{I}$ be the set of reticulation vertices connected to *v *by auxiliary edges. We say that a *tree path p*(*v, v'*) from a vertex *v *to a vertex *v' *exists if *v' *is a descendant of *v *and every edge in *p*(*v, v'*) is either a tree- or auxiliary-edge. Furthermore, we say that a reticulation *r *is *easily reachable *from a vertex *v *if a tree path *p*(*v*, $v_{p}^{r}$) exists. Finally, let *R*_*v *_be the set of all reticulations that are easily reachable from the vertex *v*.

The set *R*_*v *_can be divided into those reticulations *r *for which *v *= *lsa*(*r*), which we will again denote by $R_{v}^{G}$; *v *is a descendant of *lsa*(*r*), denoted by $R_{v}^{D}$; and *v *is an ancestor of *lsa*(*r*), denoted by $R_{v}^{A}$. If *v *is the root, $R_{v}^{D}$ is empty. The set $R_{v}^{D}$ can be divided further. Since for a reticulation *r *in $R_{v}^{D}$, the nodes directly below *lsa*(*r*) have been previously sorted, we can denote the set ${\bar{R}}_{v}^{D}$ as containing those *r *in $R_{v}^{D}$ for which *r *is sorted less than the directly descending node of *lsa*(*r*) leading via a tree path to *v*.

The aim of our optimization is to find a linear arrangement of the vertices in $V_{v}^{T}\cup V_{v}^{I}$ such that the number of reticulation edges, in the subtrees of the vertices in $V_{v}^{T}\cup V_{v}^{I}$, intersecting with tree edges is minimized. We define the *optimal linear arrangement graph OLA*^*v *^(*V*, *E*) of a vertex *v *as one that contains a vertex representative for any vertex in $V_{v}^{T}\cup V_{v}^{I}$. We add a weighted edge between any two vertices (*v*_*i*_, *v*_*k*_) in *V *and set the weight *w*_*ik *_of the edge to $\left|R_{v_{i}}^{D}\cap R_{v_{k}}^{D}\right|$. More formally written:

**Problem 1 ***With*

$$x_{ik}=\{\begin{array}{cc} \begin{array}{ll} 1 & if\;vertex\;i\;takes\;position\;k,\\ 0 & otherwise, \end{array} & \forall i,k \end{array}$$

minimize

$$\begin{array}{l} \begin{array}{l} \sum_{(i,j)\in E}w_{ij}x_{ik}x_{jl}\left|k-l\right|+\sum_{i\in V}\left|R_{v_{i}}^{D}\cap {\bar{R}}_{n}^{D}\right|x_{ik}\left|k\right|+\\ \sum_{i\in V}\left|R_{v_{i}}^{D}\cap \left(R_{n}^{D}\backslash {\bar{R}}_{n}^{D}\right)\left|x_{ik}\right|\left|V\right|-k\right| \end{array}\\ \begin{array}{ccc} subject\;to & \sum_{i\in (V)}x_{ik}=1 & \begin{array}{cc} and & \begin{array}{cc} \sum_{k\in \{1,...,\left|V\right|\}}x_{ik}=1, & \forall i,k \end{array} \end{array} \end{array} \end{array}$$

The optimal linear arrangement problem is known to be *hard *[24]. Nevertheless, this arrangement problem is in general much smaller than the complexity of minimizing all crossing edges at once. Interestingly, a couple of additional restrictions exist that we may apply to the ordering, leading to a "greedy" solution that works well in most cases. One restriction that we can place upon the structure is that for any reticulation *r*, the position in the ordering should be between $v_{p}^{r}$ and $v_{q}^{r}$. Consequently, we should place $v_{p}^{r}$ and $v_{q}^{r}$ before we place *r*.

Another restriction we can place is a consequence from the dependency of the reticulations upon each other. For any pair of reticulation *r, r' *in $R_{v}^{G}$ we say that *r *is less than *r' *if and only if a tree path *p*(*r*, $v_{p}^{r^{\prime }}$) exists. To meet the first restriction we have to place *r *before we can place *r'*. The graph that can be constructed from the relations between the reticulations must be cycle free, since the reticulation network is cycle free. Consequently, we can use a standard topological sorting algorithm to obtain a linear ordering *Ord*_*l *_($R_{v}^{G}$) for the reticulations in $R_{v}^{G}$.

**Figure 1:**
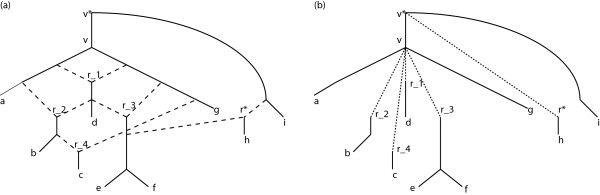
**Example of the layout optimization**. The figure on the left side shows an explicit phylogenetic network. The reticulation edges of the network are shown as dashed lines. Removing the reticulation edges and integrating the auxiliary edges into the network leads to a tree structure, as shown on the right-hand side of the figure (auxiliary edges are drawn as dashed lines). The set of easily reachable edges *R*_*v *_of the node *v *contains the reticulations *r*1, *r*2, *r*3, *r*4 and r*. The set $R_{v}^{D}$ only contains r* and is equal to ${\bar{R}}_{v}^{D}$. The placement of r* has cost 1 since the reticulation edges only cross the edge that leads to leaf *g*. The placement of *r*4 has cost 2, since the reticulation edges to the right crosses *r*1 and *r*3.

The optimization algorithm iterates through the ordering and at each reticulation *r *it first places $v_{p}^{r}$ and $v_{q}^{r}$, if necessary, and then *r*. If all reticulation are placed, the algorithm places all descending tree edges that have not yet been placed. At each placement, the algorithm positions the vertex at the position that minimizes the score given in Problem 1. After all nodes have been placed in the linear arrangement, the result is returned to the main method. An Example of the optimization procedure can be seen in Figure 1.

#### Optimal placement for reticulation vertices

Having calculated the angle and optimal arrangement for each edge, we have to place the vertices. Tree vertices can be placed in the same way as in the standard equal angle algorithm. But since auxiliary edges do not come with a given length, we have to calculate an optimal placement for each of the reticulation vertices. Such a placement has to incorporate the conditions of the equal angle algorithm, otherwise we might face unnecessary crossings between edges. Note that there are two cases for which we have to consider different placement methods. In the first case, we have a reticulation *r *where the nodes $v_{p}^{r}$ and $v_{q}^{r}$ are both different from *lsa*(*r*), and in the second case, one of them is equal to *lsa*(*r*).

**Figure 2:**
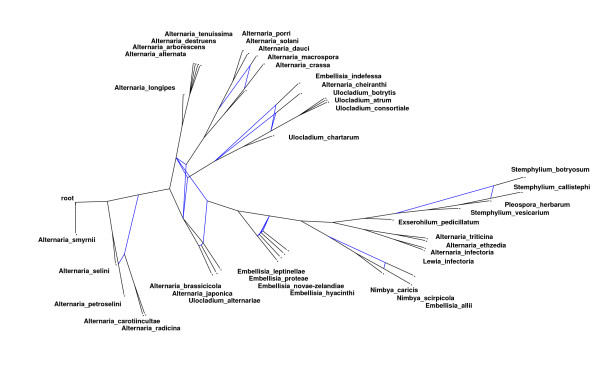
**Example of the drawing algorithm**. The figure shows the drawing of a reticulation network that we recently published [20] which is based on three gene trees described in [28].

In both cases, we place the reticulation vertex *r *on the bisector of the sector assigned to its auxiliary edge. In the first case, the distance between *r *and *lsa*(*r*) should be larger than the minimum distance between *lsa*(*r*) and the line *l*(*v*_*p*_, *v*_*q*_), indicating that *r *is a descendant of *v*_*p *_and *v*_*q*_. In other words, we assume the angles *v*_*q*_*v*_*p*_*r *and *v*_*p*_*v*_*q*_*r *are positive. In the second case, we assume that *v*_*q *_is equal to *lsa*(*r*). We first calculate the point on the bisector *r*_*t *_that has the same distance to *lsa*(*r*) as *v*_*p *_and than ensure that the angle between *r*_*t*_*v*_*p*_*r *is positive. We added an option to the algorithm so that the user can specify the (maximum) value of this angle; the standard value is 15°. An example of the drawing algorithm can be seen in Figure 2.

## Implementation

We started to integrate explicit phylogenetic networks into SplitsTree in our RECOMB 2005 article [6]. Originally, such methods were squeezed into the existing data structures within SplitsTree. SplitsTree itself is built around a group of core classes, each one representing a different type of information. The standard file format of SplitsTree is the *Nexus *[25] file format and each core class has its own Nexus representation. Consequently, developing a Nexus representation of explicit phylogenetic networks is essential for the integration of these into SplitsTree.

To build a Nexus representation for an explicit phylogenetic network, one needs to find an efficient way to present it as a string. We decided to use a version of the *extended Newick *(eNewick) [26] format. In general, the eNewick format allows labels to be present up to two times within the network. A label is allowed to appear once as a leaf and once as an internal label. Whenever a label occurs twice, the leaf is identified with the internal vertex, thus providing a network with vertices of indegree two. A lot of research has lately been focused on proving some interesting decomposition theorems [6,16,20] for explicit phylogenetic networks. The general motivation of these theorems is that the calculation of a reticulate network, with a minimal number of reticulation events, from some given information is hard [27]. The idea is to decompose each network into its two-connected components and to calculate the minimal solutions of each two-connected component separately.

Following the idea of decomposing explicit phylogenetic networks, each two-connected component may have several solutions and the possible combinations of these solutions grows exponentially, which is a problem if the number of two-connected components is large. Consequently, we decided that the Nexus representation of the network needs to reflect the two-connected components within it.

Note that any reticulate network contains either a two-connected component or a tree like element, that contains the root. We call this particular element the *root component*. The two-connected components are called *netted components*, and for each netted component, a number of solutions may exist. Any connected component that is not a two-connected component is a *tree component*. Each tree component may appear more than once within the possible configurations. The possible combinations of these three basics elements is left to the user.

**Figure 3:**
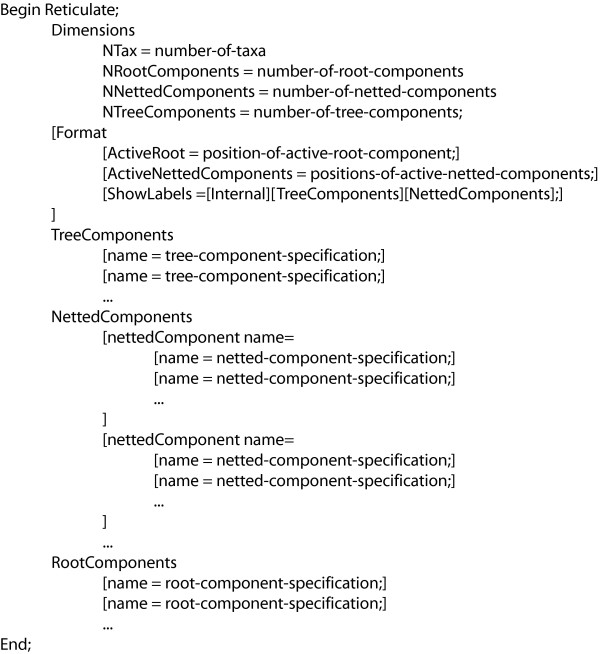
**Reticulate Nexus Block Schematic**. Shown is a schematic of the Reticulate nexus block as it is implemented in SplitsTree. The block is divided into three parts: the *Dimensions *contains all information about the dimensions of the reticulate network; the *Format *is an optional element that describes the configuration of the reticulate network; and the *TreeComponents*, *NettedComponents *and *RootComponents *contain the string representation of the reticulate network.

We now describe a Nexus notation for explicit phylogenetic networks, the schematic of this notation is shown in Figure 3. In general, one needs to save the components containing the root in the *RootComponents *section. Any such string should either be formated in standard eNewick, in Newick format where any two leaves with the same label are labeled with the name of a tree component, or in Newick format where at least one leaf is labeled with the name of a netted component.

The *NettedComponents *section contains a list of all two-connected components. Each one must be identified by a unique name and there must be at least one string representation given for each. Any such string must either be formated in eNewick, or in Newick format where any two leaves with the same label are labeled with the name of a tree component.

Finally, the *TreeComponents *section contains a list of uniquely named strings in Newick format, where leaves can be labeled with the name of netted components.

## Conclusion

In this article we presented a new algorithm for the visualization of explicit phylogenetic networks. The algorithm is a generalization of the well known equal angle algorithm and can be used to adapt most known phylogenetic tree drawing algorithm to the task of drawing reticulate networks. Moreover, we have described a datastructure and file format for representing reticulate networks in a way that reflects the structural properties of the networks.

Our implementation of these results in the popular SplitsTree software will make them accessible to biologists and other researchers that are interested in using such networks.

## Availability and requirements

• **Project name: **Drawing Phylogenetic Networks

• **Project home page: **http://www.SplitsTree.org

• **Operation system(s): **Platform independent

• **Programming language: **Java

The extensions to SplitsTree 4 are freely available for users of the application. SplitsTree 4 can be downloaded from the projects home page. Using the application is free.

## Authors' contributions

TK designed the algorithm and the integration into SplitsTree. TK and DH implemented the algorithm and the integration. TK and DH wrote the manuscript for the article.
